# Human papillomavirus prevalence in women attending routine cervical screening in South Wales, UK: a cross-sectional study

**DOI:** 10.1038/sj.bjc.6604748

**Published:** 2008-10-28

**Authors:** S Hibbitts, J Jones, N Powell, N Dallimore, J McRea, H Beer, A Tristram, H Fielder, A N Fiander

**Affiliations:** 1 Department of Obstetrics & Gynaecology, School of Medicine, Cardiff University, Heath Park, Cardiff CF14 4XN, UK; 2 Department of Pathology, Royal Gwent Hospital, Newport, NP20 2UB, UK; 3 Department of Cytology, Llandough Hospital, Cardiff and Vale NHS Trust, CF64 2XX, UK; 4 Screening Services, Breast Test Wales, 18 Cathedral Rd, Cardiff CF11 9LH, UK

**Keywords:** HPV prevalence, cervical cancer, prophylactic HPV vaccine ‘Cervarix™’, single and multiple HR cases

## Abstract

In this cross-sectional population-based study we determine human papillomavirus (HPV) prevalence in South Wales to provide comprehensive baseline data for future assessment of the impact of prophylactic HPV vaccination and to help inform future screening strategies. Liquid-based cytology samples from women attending routine cervical screening were collected (*n*=10 000: mean age 38 years, 93% cytology negative, and 64.8% from the 50% least deprived LSOA according to social deprivation score (SDS)). High-Risk (HR) and Low-Risk HPV screening was performed using HPV PCR-EIA with genotyping of HR positives and data correlated with age, SDS and cytology. Overall HPV prevalence was 13.5% (9.3% age standardised) and the most frequent HR types were HPV 16, 31, 18 and 58. In HR HPV-positive cases 42.4% had a single HR type and they were predominant in women with severe cytological abnormalities. Here, 66% of all HR HPV cases were in women aged 30 years of age or less and SDS had no significant effect on HPV status. HPV prevalence increased significantly with degree of dyskarosis from 7% in cytology negative samples to 80% in samples with severe cytological abnormalities (*P*-value <0.0001). Overall, 46% of HR HPV cases were positive for the two HR types targeted by the prophylactic vaccines (HPV 16 and HPV 18). The data presented represents the largest type-specific investigation of HPV prevalence in an unselected UK population.

Human papillomavirus (HPV) infection is the essential underlying cause of cervical cancer, found in 99.7% of invasive cervical carcinomas (Walboomers *et al*, 1999; Muñoz, 2000; Böhmer *et al*, 2003). HPV is a common cause of infection, but most of these are transient and do not result in cancer (Koutsky, 1997). Worldwide type-specific HPV prevalence in women with normal cytology is estimated to be 10.4%, highest in Africa (22.1%) and lowest in Asia (8.0%) (reviewed in de Sanjosé *et al*, 2007). Prevalence of HPV, type-specific distribution and cervical cancers vary between different regions and the five most common high-risk (HR) types are HPV 16, 18, 31, 58 and 52 with HR types 16 and 18 accounting for more than 70% of cervical cancer cases worldwide (Bosch *et al*, 1995; Clifford *et al*, 2005a, 2005b; Smith *et al*, 2007).

Multiple HR types occur in up to 43% of HR HPV-positive women and to date no association has been found between multiple HR types and progression of cervical neoplasia (Cuschieri *et al*, 2004). In contrast, clonal expansion of a single integrated HR type and cervical neoplasia development has been reported (Vinokurova *et al*, 2005). Social deprivation has been associated with cervical cancer incidence, but other co-factors such as age, smoking, education and reduced participation in screening also require consideration (de Sanjosé *et al*, 1997; Krieger *et al*, 1999; Parikh *et al*, 2003).

There are two licensed prophylactic vaccines available, Cervarix™ (GlaxoSmithKline, Middlesex, UK) and Gardasil (Merck, Hertfordshire, UK), both active against HR HPV types 16 and 18 (Paavonen *et al*, 2007; Joura *et al*, 2007). Prophylactic HPV vaccination represents a major public health breakthrough and has the potential to prevent 70–75% of cervical cancers in women vaccinated before contact with the virus, that is, before sexual debut (Harper *et al*, 2004; Villa *et al*, 2005). In October 2007, recommendations were announced that all 12–13 year old girls should be offered the vaccine and Cervarix™ will be implemented within the UK from September 2008 (DoH News, 2008 and WAG News, 2007). Current cervical screening relies on cervical cytology to identify women at risk of having ‘precancerous’ cervical intraepithelial neoplasia and this process is estimated to save around 4500 lives per year in the UK (Peto *et al*, 2004). Following implementation of HPV vaccination, the positive predictive value of cervical cytology will fall alongside the reduction in cytological abnormalities. Consequently current screening strategies will become less effective necessitating a change in screening modality. HPV typing and biomolecular marker assays have the potential to replace cervical cytology provided they demonstrate clinical utility and cost-effectiveness and there are a number of ongoing HPV trials within Europe investigating strategies to address these issues (Cuzick *et al*, 2003; Cotton *et al*, 2006; Coupé *et al*, 2008; Sargent *et al*, 2008).

We aimed to extend preliminary findings (Hibbitts *et al*, 2006) and determine the prevalence of HPV among women attending routine cervical screening and thereby provide baseline data for the future assessment of the impact of prophylactic HPV vaccination in South Wales. The findings represent the largest type-specific investigation of HPV infection in an unselected UK population (*n*=10 000; 20–65 years).

## Materials and methods

10 000 consecutive, anonymous, residual liquid-based cytology (LBC) screening samples were collected in collaboration with Cervical Screening Wales over a 5-month time period in 2004 (non-probability convenience sample). This method included all women attending for routine cervical screening within the catchment area of Llandough Hospital Cytology Laboratory and constitutes a representative sample of the screening population in South Wales. Age, cytology result and Social Deprivation Score (SDS) were provided for each specimen and inadequate cytology samples or those from colposcopy clinics were excluded. The study was approved by South Wales Local Research Ethics Committee.

The LBC samples (Thinprep) were processed by the Cytology Laboratory according to the British Society of Clinical Cytology guidelines (NHS Cancer Screening Programmes, 2004). Residual cell pellets were re-suspended in the alcohol-based liquid and processed by the HPV Laboratory, Cardiff University. Each LBC sample was washed and re-suspended in 1 ml 10 mM Tris pH 7.4. 10 *μ*l of recombinant proteinase K was added to a 100 *μ*l cell suspension from each sample, incubated at 56°C for 2 h, followed by 100°C for 10 min. Samples were refrigerated (4°C) for 10 min, centrifuged at 13 000 r.p.m. for 10 min and the supernatant transferred into a 96-well plate. A PCR for the human *β*-globin gene was performed on every DNA sample to determine extraction efficiency as described earlier (Hibbitts *et al*, 2006). The GP5/GP6+ HPV PCR-ELISA method of Jacobs *et al* (1997) was performed on all specimens in a 96-well format with minor modifications. Samples were processed in batches of 96 to increase throughput and a two-tier method applied: (i) An initial PCR-ELISA with a cocktail of HR or LR type-specific probes; (ii) A second PCR-ELISA of all HR-positive samples with genotyping using 14 individual HR HPV probes: HPV 16; 18; 31; 33; 35; 39; 45; 51; 52; 56; 58; 59; 66 and 68. The initial ‘HR/LR’ and subtyping PCR was performed in a final volume of 25 *μ*l and 100 *μ*l respectively. PCR cycling conditions were 94°C 4 min, 40 cycles of 94°C 30 s, 40°C 90 s, 72°C 60 s followed by 72°C 4 min. Positive (CaSki) and negative (water) DNA extraction, PCR and ELISA controls were included for every 94 samples. Re-analysis of 10% of all samples processed was undertaken for internal quality assessment purposes. Results were analysed in an automated Excel worksheet and raw data from each reading at each time point pasted into a specific position in the datasheet and results are then given as positive (1) or negative (0). The negative extraction control included in every 96-well plate serves as the background reading for which all the other results are compared. A positive result is equivalent to three times background.

### Statistical analysis

Statistical analysis was performed on samples that complied with the following inclusion criteria: *β*-globin PCR positive; complete information available on age, cytology result and SDS; within the screening age group 20–65 years. The HR HPV prevalence was age-standardised using the European Standard Population and the age-standardised rate calculation ∑ Si ria/∑ Si (Breslow and Day, 1987; Waterhouse, 2003). Social deprivation was estimated by linking postcodes to the Welsh Index of Multiple Deprivation which describes levels of deprivation across Wales with higher scores indicating greater deprivation (National Assembly for Wales, 2005). The overall deprivation scores in Wales range from 78.9 to 1.4. Geographical units are termed Lower Layer Super Output Areas (LSOA) and they are graded according to SDS as follows: 10% most deprived (41.91–78.9 SDS); 10–20% most deprived (32.71–41.9 SDS); 20–30% most deprived (26.31–32.70 SDS); 30–50% most deprived (17.91–26.30 SDS); 50% least deprived LSOA in Wales (1.4–17.9 SDS). The *χ*^2^ test was used to calculate a *P*-value and 95% confidence intervals (CI) were calculated where appropriate. All HR HPV-positive cases were differentiated into those with a single HR type and those with multiple HR types.

## Results

The mean age was 38 years with 29.4% aged between 20–29 years, 25.8% 30–39 years, 23.4% 40–49 years, 15.8% 50–59 years and 5.6% over 60 years old. In this study population, 93% were cytology negative, 4.7% had borderline changes and 1.4, 0.5 and 0.52% had mild, moderate and severe dyskaryosis respectively. The distribution of samples according to SDS based on the LSOA ranking system was 9.5% from the 10% most deprived; 5.6% from the 10 to 20%; 7.4% from the 20 to 30%; 12.6% from the 30 to 50%; and 64.8% from the 50% least deprived LSOA in Wales.

Of the 10 000 samples processed, 921 did not meet the inclusion criteria and full statistical analysis was performed on the remaining 9079 samples. Internal quality assessment indicated 93% reproducibility for *β*-globin-positive DNA extraction controls and 96% reproducibility for HR and LR HPV-positive results. Here, 1015 HR HPV-positive and 209 LR HPV-positive cases were identified. In the 1015 HR HPV-positive cases there were 2007 HR infections and 157 were also positive for LR HPV. Overall HPV prevalence was 13.5% (*n*=1224; 95% CI 11.3–15.9%) with an age standardised HPV prevalence in the South Wales population of 9.3%.

**Figure 1:**
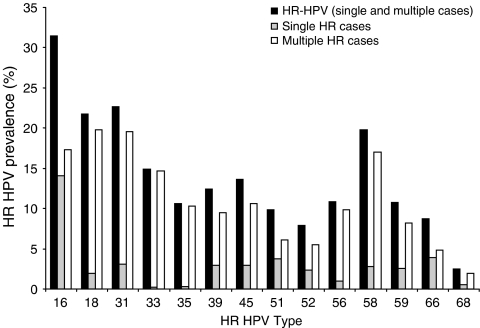
Cross-sectional overview of HR HPV type distribution in South Wales. The total number of HR HPV cases with single HR and multiple HR types in each genotype calculated as a percentage of the total number of positive cases (*n*=1015). The HR HPV prevalence of each type in order of predominance was as follows: HPV 16 (*n*=319 31.4%), HPV 31 (*n*=230 22.6%), HPV 18 (*n*=221 21.7%), HPV 58 (*n*=201 19.8%), HPV 33 (*n*=151 14.9%), HPV 45 (*n*=138 13.6%), HPV 39 (*n*=126 12.4%), HPV 56 (*n*=110 10.8%), HPV 59 (*n*=109 10.7%), HPV 35 (*n*=108 10.6%), HPV 51 (*n*=100 9.8%), HPV 66 (*n*=89 8.8%), HPV 52 (*n*=80 7.9%) and HPV 68 (*n*=25 2.5%). Single HR HPV type distribution (in order of predominance): HPV 16 (*n*=143 14.1%), HPV 66 (*n*=40 3.9%), HPV 51 (*n*=38 3.7%), HPV 31 (*n*=31 3.1%), HPV 45 (*n*=30 3.0%), HPV 39 (*n*=30 3.0%), HPV 58 (*n*=28 2.8%), HPV 59 (*n*=26 2.6%), HPV 52 (*n*=24 2.4%), HPV 18 (*n*=20 2.0%), HPV 56 (*n*=10 1.0%), HPV 68 (*n*=5 0.5%), HPV 35 (*n*=3 0.3%), and HPV 33 (*n*=2 0.2%). Multiple HR HPV type distribution: HPV 18 (*n*=201 19.8%), HPV 31 (*n*=199 19.6%), HPV 16 (*n*=176 17.3%), HPV 58 (*n*=173 17.0%), HPV 33 (*n*=149 14.7%), HPV 45 (*n*=108 10.6%), HPV 35 (*n*=105 10.3%), HPV 56 (*n*=100 5.5%), HPV 39 (*n*=96 9.5%), HPV 59 (*n*=83 8.2%), HPV 51 (*n*=62 6.1%), HPV 52 (*n*=56 5.5%), HPV 66 (*n*=49 4.8%), and HPV 68 (*n*=20 2.0%).

**Figure 2:**
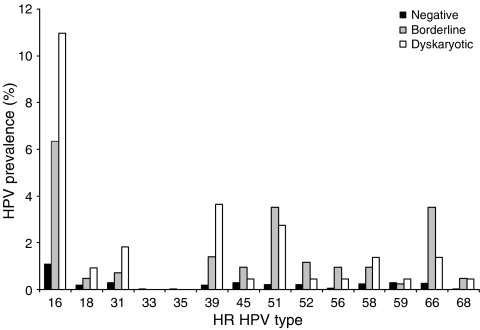
HR HPV type-specific prevalence by cytology grade in cases with a single HR type (*n*=430). HPV prevalence calculated as a percentage of the total number of women in the study population classified as negative (*n*=8434), borderline (*n*=426) and dyskaryotic (*n*=219) cytology.

In HR HPV-positive cases (*n*=1015) the most prevalent HR genotypes detected were 16 (31.4%), 31 (22.6%), 18 (21.7%) and 58 (19.8%). The proportion of HR HPV cases attributable to types included in currently licensed prophylactic HPV vaccines (HPV 16 and HPV 18) was 46% overall (*n*=471 out of 1015) and 70% (*n*=28 out of 40) for cases with severe cytological abnormalities. Here, 42% (*n*=430 out of 1015; 95% CI 39.4–45.5%) of HR HPV-positive cases had an infection with a single HR type and in these women HPV 16 was the predominant genotype (*n*=143 out of 430). The remaining 58% (*n*=585 out of 1015; 95% CI 54.5–60.6%) had multiple HR types and HPV 18 was the most predominant type detected (*n*=201 out of 585). The prevalence of each HPV genotype in cases with a single HR infection was compared with cases containing multiple HR types and no statistically significant differences were found for HPV 16, 51 or 66 (*χ*^2^ test; *P*=0.2594, *P*=0.3461 and *P*=0.6142 respectively). All other genotypes were statistically significantly more common in cases with multiple HR types compared with those with a single HR type (Figure 1). In cases with a single HR type the genotype distribution in each cytology grade (negative, borderline and dyskaryotic) was assessed. HPV 16 was the only genotype significantly more prevalent in cases with dyskaryosis (mild, moderate or severe) compared with borderline and negative cases (Figure 2; *χ*^2^ test; *P*=0.0395 and *P*>0.0001 respectively).

**Figure 3:**
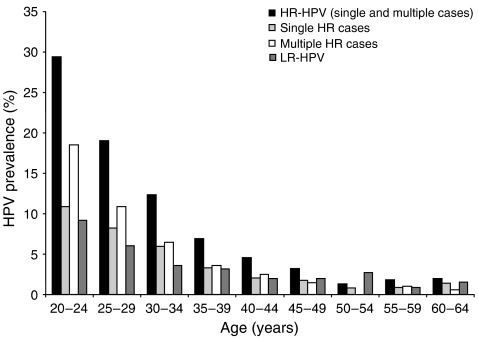
HPV prevalence distribution by age. The total number of HR HPV, single HR, multiple HR, and LR HPV cases in each age group was calculated as a percentage of the total number of women in the study population (9079) classified as 20–24 years (*n*=1578), 25–29 years (*n*=1096), 30–34 years (*n*=1164), 35–39 years (*n*=1175), 40–44 years (*n*=1157), 45–49 years (*n*=966), 50–54 years (*n*=739), 55–59 years (*n*=694) and 60–64 years (*n*=510).

Here, 66% of all HR and 57.6% of LR HPV cases were found in women aged 30 years or less and the prevalence of HR cases decreased progressively with age from 29.4% at 20–24 years to 1.4–2% at ages 50–65 years (Figure 3). In concordance, the majority of all single HR cases (*n*=262 out of 430) and cases with multiple HR types (*n*=411 out of 585) were found in women aged 30 years or less. Cases with multiple HR types were more common than single HR cases in all women upto 44 years and this was statistically significant in women aged 30 or less (20–24 years *P*<0.0001; 25–29 years *P*=0.0349).

HPV prevalence by cytology grade was calculated as a percentage of the total number of women in the study population classified as negative (*n*=8434), borderline (*n*=426), mild (*n*=126), moderate (*n*=43) and severe (*n*=50). There was a statistically significant increase in the proportion of HR HPV-positive cases from 7% (*n*=590 out of 8434) in cytology negative samples up to 80% (*n*=40 out of 50) with severe dyskaryosis (*P*<0.0001). There was a limited variation between total HR HPV prevalence in cases with borderline, mild, moderate and severe dyskaryosis because of the many cases with multiple HR types in all grades with abnormal cytology. In contrast, cases with a single HR type predominated in women with severe dyskaryosis and this was significant relative to their prevalence in negative, borderline, mild and moderate cytology samples (*P*<0.0001; *P*=0.0013; *P*=0.0100; *P*=0.0135 respectively). Severe cytological abnormalities were detected in 50 cases and 40 out of 50 were also HR HPV positive: 21 out of 40 cases contained a single HR HPV infection 14 of which were positive for HPV 16 and in the remaining 19 out of 40 cases with multiple HR types 11 were HPV 16 positive. Here, 25 of the 366 LR HPV cases detected in this study population had an abnormal cytology (classified as mild, moderate or severe).

**Figure 4:**
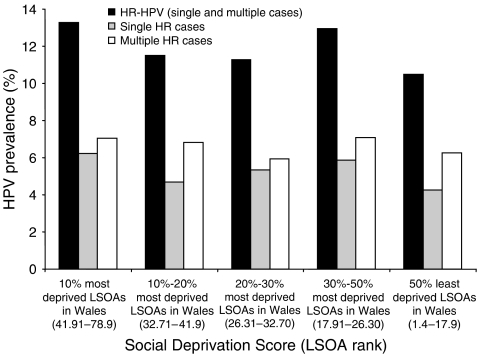
HPV Prevalence Distribution with Social Deprivation Score. The total number of HR HPV, single HR and multiple HR cases in each LSOA rank was calculated as a percentage of the total number of women in the study population (9079) classified as 10% most deprived LSOA (*n*=866), 10–20% most deprived LSOA (*n*=512), 20–30% most deprived LSOA (*n*=673), 30–50% most deprived LSOA (*n*=1144) and 50% least deprived LSOA in Wales (*n*=5884).

Most of the women in the study were from the 50% least deprived LSOA (64.8%) and the lowest HR HPV prevalence was detected in the 50% least deprived LSOA in Wales (10.5%) with the highest in the 10% most deprived LSOA (13.3%) according to SDS. However, HR HPV cases were not significantly more prevalent in any one rank (Figure 4).

## Discussion

This is the largest type-specific investigation of HPV infection in an unselected UK population to date, with an overall HPV prevalence of 13.5% (9.3% age standardised). This baseline HPV data was particularly robust for two reasons: (i) Samples were anonymous and therefore did not require consent allowing collection of consecutive samples from all women attending routine cervical screening; (ii) A two-tier typing procedure was followed in which HR-positive samples were identified and then validated with a second HPV PCR before typing for 14 individual HR HPV types, thus reducing the risk of false positive results. Stringent quality control procedures were also employed involving random retyping of 10% samples.

Within Europe, the closest comparative study was in Scotland (Cuschieri *et al*, 2004), the overall HPV prevalence being 20.5% (non age standardised), higher than in South Wales. However, differences may be partially attributable to the methodologies employed; in the Scottish study a subset of 3444 randomly selected specimens with informed consent were analysed and successful amplification with consensus primers was taken as a positive result, whereas in the present study 10 000 unselected samples were processed and only those giving two consecutive positive results were considered positive.

HR HPV conveys an increased risk of development of cervical neoplasia and in this study HR HPV prevalence increased with cytological abnormality (Bosch *et al*, 1995). A significantly higher number of cases with a single HR type had severe dyskaryosis compared with other cytology grades and no such correlation with disease progression was found in cases with multiple HR types. Others have recognised the clonal expansion of a single integrated HR type and subsequent cervical neoplasia development (Vinokurova *et al*, 2005) and our data would support this observation and suggests that there is little, if no, synergy between multiple HPV types in terms of neoplastic effect (Fife *et al*, 2001). However, the overall HPV type-specific distribution in cases with multiple HR types is not a direct reflection of that observed in cases with a single HR type, and of interest was the equivalent representation of HPV 16 (Figure 1). HPV 16 is more persistent and aggressive than other HR types (Smith *et al*, 2007) and our data suggests it is also capable of creating an environment which is permissive to secondary and/or co-infection with other HR types.

The highest HR HPV prevalence was found in women aged 30 years or less consistent with other findings (de Sanjosé *et al*, 2007) and this is associated with a higher number of recent sexual partners linked with this age group. In this study population, a high number of cases with multiple HR types (58%) were observed compared with other reports (Cuschieri *et al*, 2004). However, 70% (*n*=411 out of 585) of these women were aged 30 years or less and more recent studies have observed an increasing number of multiple infections within this age group (Sargent *et al*, 2008). Further HPV type-specific PCR sequence analysis of cases with multiple infections will be performed to confirm the QC validated results obtained utilising the GP5/GP6+ HPV PCR-EIA assay.

The use of an anonymous screening population avoids many potential sources of bias. However, it inevitably only includes people who attend for their smears and we do not have data on the HPV status of non-attendees. The reported correlation of increased cervical cancer incidence associated with social deprivation (de Sanjosé *et al*, 1997) is not reflected in our data and we observed no significant increase in HPV prevalence in women from the most deprived areas of South Wales. This may be because women from areas of social deprivation are less likely to undergo cervical screening.

We identified HPV 16, 31, 18 and 58 as the predominant HR types, which is broadly consistent with other studies (Clifford *et al*, 2005a, 2005b). Our data indicates that the prophylactic vaccination has the potential to target HR types 16 and 18 in up to 46% of women with negative cytology and 70% of cases with severe dyskaryosis in South Wales. However, additional factors need to be considered including vaccination uptake, successfully induced immunity and residual susceptibility to carcinogenic HPV types. The vaccination programme is likely to change HR HPV type-specific prevalence and there is a need to include HPV genotyping assays into cervical screening programmes to form a HPV type-specific surveillance to monitor the efficacy of prophylactic vaccination. Our data represents a baseline for future analyses in this population to monitor vaccination programme effects on the prevalence of the vaccine-targeted HR types 16 and 18, the proportion of cross-protection provided to non-vaccine HR HPV types and the issue of type-replacement. For the foreseeable future cytology will remain a key part of cervical screening, however, changes to current screening strategies are inevitable and there is only a short time frame for incorporating robust HPV typing methods into routine settings.

In summary, current prevalence of HPV infection in South Wales is 13.5% and half of all current HR HPV cases would be targeted by the prophylactic HPV vaccines.
